# Awareness, Usage and Perceptions of Doxycycline Post‐Exposure Prophylaxis (doxyPEP) for Prevention of Sexually Transmitted Infections in Australia: Insights From a National Cross‐Sectional Survey

**DOI:** 10.5694/mja2.70180

**Published:** 2026-04-14

**Authors:** Phyu Mon Latt, Ei T. Aung, Kate Maddaford, Kai J. Jonas, Christopher K. Fairley, Sarah J. Martin, Carole Khaw, Rick Varma, Caroline Thng, Manoji Gunathilake, Vincent J. Cornelisse, Haoyi Wang, Teralynn Ludwick, Ethan T. Cardwell, Michael W. Traeger, Catriona S. Bradshaw, Dash Heath‐Paynter, Benjamin Riley, Daniel Grace, Fabian Y. S. Kong, Eric P. F. Chow, the DoxyAWARE Study Group, Adam Bament, Adam Hynes, Alison Mahony, Andrew Heslop, Angus Molyneux, Belinda Wozencroft, Beng Eu, Cameron Brown, Carolyn Spivak, Catriona Ooi, Clara Tuck Meng Soo, Donna Keeley, Elaine Sung, Fergus McCabe, Gordon Stacey, Jen Johnson, Karen Bonte, Lucy Watson, Luke Coffey, Marcus Shaw, Melissa Warner, Michelle Doyle, Samantha Johnson, Sara Graham, Timothy Krulic, Vikram Rai, Wee‐Sian Woon

**Affiliations:** ^1^ Monash University Melbourne Victoria Australia; ^2^ Melbourne Sexual Health Centre, Bayside Health Melbourne Victoria Australia; ^3^ Maastricht University Maastricht the Netherlands; ^4^ Canberra Sexual Health Centre, Canberra Health Services Canberra Australian Capital Territory Australia; ^5^ Australian National University Canberra Australian Capital Territory Australia; ^6^ Adelaide Sexual Health Centre, Royal Adelaide Hospital Adelaide South Australia Australia; ^7^ Adelaide University Adelaide South Australia Australia; ^8^ Sydney Sexual Health Centre, South Eastern Sydney Local Health District Sydney New South Wales Australia; ^9^ Kirby Institute UNSW Sydney Sydney New South Wales Australia; ^10^ Sexual Health Services, Gold Coast Health Gold Coast Queensland Australia; ^11^ Institute for Biomedicine and Glycomics Griffith University Gold Coast Queensland Australia; ^12^ Centre for Disease Control, Northern Territory Department of Health Darwin Northern Territory Australia; ^13^ Mid North Coast Local Health District Port Macquarie New South Wales Australia; ^14^ Centre for Epidemiology and Biostatistics University of Melbourne Melbourne Victoria Australia; ^15^ Burnet Institute Melbourne Victoria Australia; ^16^ Health Equity Matters Sydney New South Wales Australia; ^17^ Australasian Society for HIV, Viral Hepatitis and Sexual Health Medicine Sydney New South Wales Australia; ^18^ University of Toronto Toronto Canada

**Keywords:** antimicrobial resistance, bacterial infections, doxycycline post‐exposure prophylaxis, gay, bisexual and men who have sex with men, sexually transmitted diseases

## Abstract

**Objective:**

To examine the awareness, usage and perceptions of doxycycline post‐exposure prophylaxis (doxyPEP) for sexually transmitted infection (STI) prevention among gay and bisexual men and transgender (trans) and gender diverse people in Australia.

**Design:**

Cross‐sectional online survey.

**Setting, Participants:**

National multi‐site survey in Australia from 1 July 2024 to 30 November 2024, recruiting from 13 sexual health and community clinics, 6 general practices, social media, dating applications, and university portals. Gay and bisexual men and trans and gender diverse people aged ≥ 18 years living in Australia were included in the study.

**Main Outcome Measures:**

DoxyPEP awareness, ever use, recent use (past 12 months), dosage regimens, sourcing methods and planned future use.

**Results:**

Among 2095 participants, half (1080/2095, 51.6%) had heard of doxyPEP. Of those aware, 323/1080 (29.9%) had ever used doxyPEP, and 306/1080 (28.3%) were recent users. DoxyPEP awareness and usage varied by HIV status and pre‐exposure prophylaxis (PrEP) use (*p* < 0.0001). Nearly two‐thirds of users had taken the recommended 200 mg within 72 h after sex (205/323, 63.5%). Among recent users, 29/306 (9.5%) reported recent syphilis diagnoses, and 85/306 (27.8%) had ≥ 2 STI diagnoses in the past 12 months. Of those who had ever used doxyPEP, 135/323 (41.8%) obtained prescriptions from clinicians, 17/323 (5.3%) obtained it online, and 28/323 (8.7%) purchased it in person overseas without a prescription. Of those aware of doxyPEP, 490/1080 (45.4%) planned to use doxyPEP in the next 12 months, primarily to prevent chlamydia (460/490, 93.9%), gonorrhoea (422/490, 86.1%) or syphilis (386/490, 78.8%). Some intended to prevent *Mycoplasma genitalium* (92/490, 18.8%) or mpox (36/490, 7.4%). Among non‐users, 306/756 (40.5%) worried about antibiotic resistance.

**Conclusions:**

DoxyPEP use was happening quickly but often involved non‐recommended regimens and unsupervised sourcing. Urgent educational interventions and improved clinical access are needed for safe implementation.

## Plain Language Summary

**The Known:** Doxycycline post‐exposure prophylaxis (doxyPEP) reduces bacterial sexually transmitted infections by 53% in men who have sex with men and in transgender women. A 2023 Australian consensus statement recommends doxyPEP primarily for those with recent syphilis or multiple diagnoses.

**The New:** In a national survey of 2095 participants, 51.6% had heard of doxyPEP, of which one‐third had ever used it. One‐third used non‐recommended regimens, one in seven sourced it without a prescription, and situational risk factors mainly drove usage.

**The Implications:** Clinicians and health departments should prioritise education, supervised access and stewardship to align community practice with guidance and strengthen antimicrobial resistance monitoring.

## 1 Introduction

As rates of sexually transmitted infections (STIs) increase globally, new prevention interventions are urgently needed [1]. Following successful human immunodeficiency virus (HIV) pre‐exposure prophylaxis (PrEP) implementation [2], studies have evaluated whether doxycycline taken prophylactically post‐exposure (doxyPEP) or pre‐exposure (doxyPrEP) can reduce bacterial STIs, namely syphilis, chlamydia and gonorrhoea [3]. The recommended doxyPEP regimen involves taking 200 mg of doxycycline within 72 h after sexual exposure [4]. A systematic review and meta‐analysis published in 2025 summarised four studies and reported 53% (hazard ratio [HR], 0.47; 95% confidence interval [CI], 0.38–0.60) overall STI reduction among men who have sex with men (MSM) and transgender (trans) women, with greater effectiveness against chlamydia and syphilis (78% [relative risk (RR), 0.22; 95% CI, 0.13–0.38] and 77% [RR, 0.23; 95% CI, 0.13–0.41] respectively) than gonorrhoea (22% [RR, 0.78; 95% CI, 0.65–0.94]) [5]. There is limited evidence supporting the effectiveness of doxyPEP in cisgender women (potentially due to low drug adherence) [6], but small studies in Japanese sex workers reported that doxyPrEP was effective in reducing the incidence of chlamydia and syphilis, but not gonorrhoea [7].

Implementation of doxyPEP has been endorsed by several countries, such as the United States [8], the United Kingdom [9] and Australia [4]. Previous studies from England, Australia, the Netherlands and the United States have estimated that about 8%–21% of MSM have used doxyPEP [10, 11, 12, 13, 14, 15]. Among countries that have not fully endorsed doxyPEP, one of the main concerns is that the widespread use of doxycycline may potentially increase antimicrobial resistance in STI pathogens (e.g., *Neisseria gonorrhoeae* ) [16] and bystander commensal organisms (e.g., *Staphylococcus aureus* ) [17]. *N. gonorrhoeae* resistance is of global concern, as resistance to the last remaining effective treatment, ceftriaxone, is increasing globally [16, 18], and *S. aureus* is among the top six pathogens responsible for the majority of deaths due to antimicrobial resistance in 2019 [19]. Furthermore, studies have reported delayed syphilis diagnoses among doxyPEP users [20, 21], suggesting a potential indirect impact of using doxyPEP that may complicate timely diagnosis and treatment, particularly when doxyPEP is recommended for primary syphilis prevention.

Although a previous Australian community‐based study has demonstrated high acceptability of doxyPEP (75.8%) among gay and bisexual men [11], this was conducted before the Australian consensus statement was published in September 2023 [4]. It recommends doxyPEP primarily for syphilis prevention among gay, bisexual and other men who have sex with men (GBMSM), with the eligibility criteria including recent syphilis diagnosis, two or more recent bacterial STI diagnoses, upcoming periods of heightened STI risk (such as attendance at sex events), concurrent male and female sexual partners or presentation for HIV post‐exposure prophylaxis [4].

Limited data exist on actual implementation patterns following the release of the consensus statement, including everyday usage practices, adherence to recommended regimens and alignment between policy recommendations and community practice. With the rapid rollout of doxyPEP, there is an urgent need to examine the awareness, usage and perceptions of using doxyPEP in the community. Understanding how the community perceives the benefits and harms of using doxyPEP is important to optimise clinical care and management and to support a safe and effective use of doxyPEP.

## 2 Methods

This study is reported according to the CROSS (Consensus‐Based Checklist for Reporting of Survey Studies) guidelines [22] (Section S1).

### 2.1 Study Design and Participants

We conducted an online, anonymous survey hosted on Qualtrics software (Provo, US) between 1 July and 30 November 2024. Individuals were eligible if they met four criteria: (i) were aged ≥ 18 years; (ii) identified as a man (cisgender or trans), trans woman, or non‐binary person who has sex with men; (iii) were currently living in Australia; and (iv) had at least Year 10 level English (able to read most newspaper articles). No prior doxyPEP knowledge was required. Participants provided informed consent before starting and could enter a draw for one of five $200 gift vouchers.

Participants were recruited from 19 clinical sites (13 sexual health clinics and 6 general practices) in Australia (Table S1). Recruitment methods included placing flyers in the clinics and handing our business cards with a QR code to the survey links to potential eligible participants at clinical sites, targeted advertisements on dating applications (apps) and social media and word‐of‐mouth referrals through community networks.

### 2.2 Data Collection and Measures

This survey was adapted from a doxyPEP study conducted in the Netherlands [23]. It was co‐designed and substantially modified for the Australian context through consultations with sexual health researchers, epidemiologists, clinicians, pharmacists and lesbian, gay, bisexual, transgender, queer or questioning, intersex, asexual or aromantic or agender, and other diverse sexual orientations and gender identities (LGBTQIA) + and HIV community‐controlled organisations. Gender identity was self‐reported, with participants identifying as cisgender men, transgender men, transgender women or non‐binary. We use ‘trans and gender diverse’ to collectively describe transgender and non‐binary participants.

The survey assessed: (i) demographic characteristics (age, country of birth, HIV status, HIV PrEP use); (ii) doxyPEP awareness, uptake (ever use and recent use in past 12 months), dosage regimens, sourcing methods, reasons for use/non‐use and future plans; and (iii) sexual practices and STI diagnoses. Participants aware of doxyPEP ranked their top three trusted information sources from eight options and rated community benefit (0–10 scale) with an optional explanation. Data quality measures included attention checks, reCAPTCHA and honeypot questions to detect and exclude automated or inattentive responses.

### 2.3 Statistical Analyses

We used descriptive statistics to summarise participant characteristics. We report continuous variables as medians with interquartile ranges (IQRs) due to non‐normal distributions and categorical variables as frequencies and proportions. We used chi‐squared tests to compare proportions between groups, such as doxyPEP awareness by recruitment setting.

To identify factors associated with recent doxyPEP use, we conducted a multivariable logistic regression analysis among those who were aware of doxyPEP, using backward elimination (initial inclusion: *p* < 0.20; final retention: *p* < 0.05). Model fit was assessed using Hosmer–Lemeshow test and C‐statistic. We reported odds ratios (ORs) with 95% CIs from univariable analyses and adjusted odds ratios (aORs) with 95% CIs from multivariable analyses.

For open‐ended responses, we used the ‘wordcloud’ package in R and the structural topic modelling (‘stm’ package) to identify key themes and linear regression to examine associations between topic probability [24] and perceived benefit scores. Regression coefficients (*β*) represent the change in benefit score per topic; positive values indicate a higher perceived benefit (Section S1). All analyses used Stata (version 17) and R (version 4.2.1).

### 2.4 Ethics Statement

This study received ethics reviews and approvals from the Alfred Hospital Ethics Committee (#107/24), the AIDS Council of New South Wales (ACON) (#202405), Thorne Harbour Health (#THH_2024_010) and Human Research Ethics Committee of Northern Territory Health and Menzies School of Health Research (#2024‐4878) and was registered with Monash University (#48959).

## 3 Results

### 3.1 Demographic Characteristics of Study Participants

Of the 3940 individuals who initiated the survey, 1845 were excluded (Table S2), resulting in the final sample of 2095 participants. The median survey completion time was 5.6 min (IQR, 4.0–8.4).

The median age of participants was 39 years (IQR, 31–50). Most participants were cisgender men (1984/2095, 94.7%), identified as gay (1643/2095, 78.4%) and were recruited from non‐clinical settings (1427/2095, 68.1%). Most participants were HIV PrEP users (1368/2095, 65.3%), whereas 536/2095 (25.6%) were HIV‐negative non‐PrEP users, and 191/2095 (9.1%) were people living with HIV. The median number of sexual partners in the past 12 months was 12 (IQR, 5–30) (Table 1). Among all participants, 129/2095 (6.2%) reported recent syphilis diagnoses in the past 12 months, 269/2095 (12.8%) had gonorrhoea and/or chlamydia diagnoses, and 324/2095 (15.5%) had two or more STI diagnoses in the past 12 months.

**TABLE 1:** Participants' demographic characteristics.

Characteristics	Number of participants
Total number of participants	2095
Age, median (IQR)	39 (31–50)
Gender identity, *n* (%)	
Cisgender man	1984 (94.7%)
Trans man	32 (1.5%)
Trans woman	16 (0.8%)
Non‐binary person	59 (2.8%)
Prefer not to answer	4 (0.2%)
Sexual identity, *n* (%)	
Gay or homosexual	1643 (78.4%)
Straight or heterosexual	45 (2.2%)
Bisexual	283 (13.5%)
Queer	87 (4.2%)
Another term	26 (1.2%)
Prefer not to answer	11 (0.5%)
Education level, *n* (%)	
High school	336 (16.0%)
Tertiary diploma or trade certificate	396 (18.9%)
University	1363 (65.1%)
Type of recruitment, *n* (%)	
Clinical (sexual health and general practice clinics)	668 (31.9%)
Non‐clinical (social media, dating apps)	1427 (68.1%)
HIV and pre‐exposure prophylaxis use, *n* (%)	
HIV negative, not taking pre‐exposure prophylaxis	536 (25.6%)
HIV negative, taking pre‐exposure prophylaxis	1368 (65.3%)
People living with HIV	191 (9.1%)
Number of sexual partners in the past 12 months, median (IQR)	12 (5–30)
Aware of doxyPEP, *n* (%)	
Yes	1080 (51.6%)
No	939 (44.8%)
Unsure	76 (3.6%)
Sources where participants learnt about doxyPEP,^a^ *n* (%)	
Friends	431 (39.9%)
Healthcare professionals (e.g., doctors)	330 (30.6%)
HIV/LGBTIQA+ community‐controlled organisations	221 (20.5%)
Online community group or forum (e.g., Facebook)	229 (21.2%)
Online providers of doxyPEP	62 (5.7%)
Sexual partners	245 (22.7%)
Social media or news	281 (26.0%)
Other^b^	88 (8.1%)
STI diagnoses in the past 12 months, *n* (%)	
Recent syphilis diagnosis	129 (6.2%)
Gonorrhoea and/or chlamydia	269 (12.8%)
≥ 2 STI diagnoses	324 (15.5%)

Abbreviations: apps, applications; doxyPEP, doxycycline post‐exposure prophylaxis; HIV, human immunodeficiency virus; IQR, interquartile range; LGBTIQA+, lesbian, gay, bisexual, transgender, intersex, queer and asexual +; STI, sexually transmitted infection; trans, transgender.

^a^ Participants can choose more than one source. The number of participants who were aware of doxyPEP (1080) was used as the denominator to calculate the proportion.

^b^ Other sources included learning about doxyPEP on other dating app users' profiles.

### 3.2 Awareness and Information Sources

Overall, half of the participants (1080/2095, 51.6%) were aware of doxyPEP at the time of the survey (Table 1). Awareness was significantly higher among HIV PrEP users (806/1368, 58.9%) and people living with HIV (97/191, 50.8%) compared with HIV‐negative participants not using PrEP (177/536, 33.0%; *p* < 0.0001). Participants recruited from clinical settings also had significantly higher awareness than those from non‐clinical settings (403/668 [60.3%] vs. 677/1427 [47.4%]; *p* < 0.0001) (Table S3).

Among participants who had heard of doxyPEP, the most frequently reported source of information was friends (431/1080, 39.9%), but healthcare professionals were most trusted (weighted mean score = 2.8 out of 3), followed by peer community organisations (Table S4).

### 3.3 Uptake and Dosage Regimens

**FIGURE 1:**
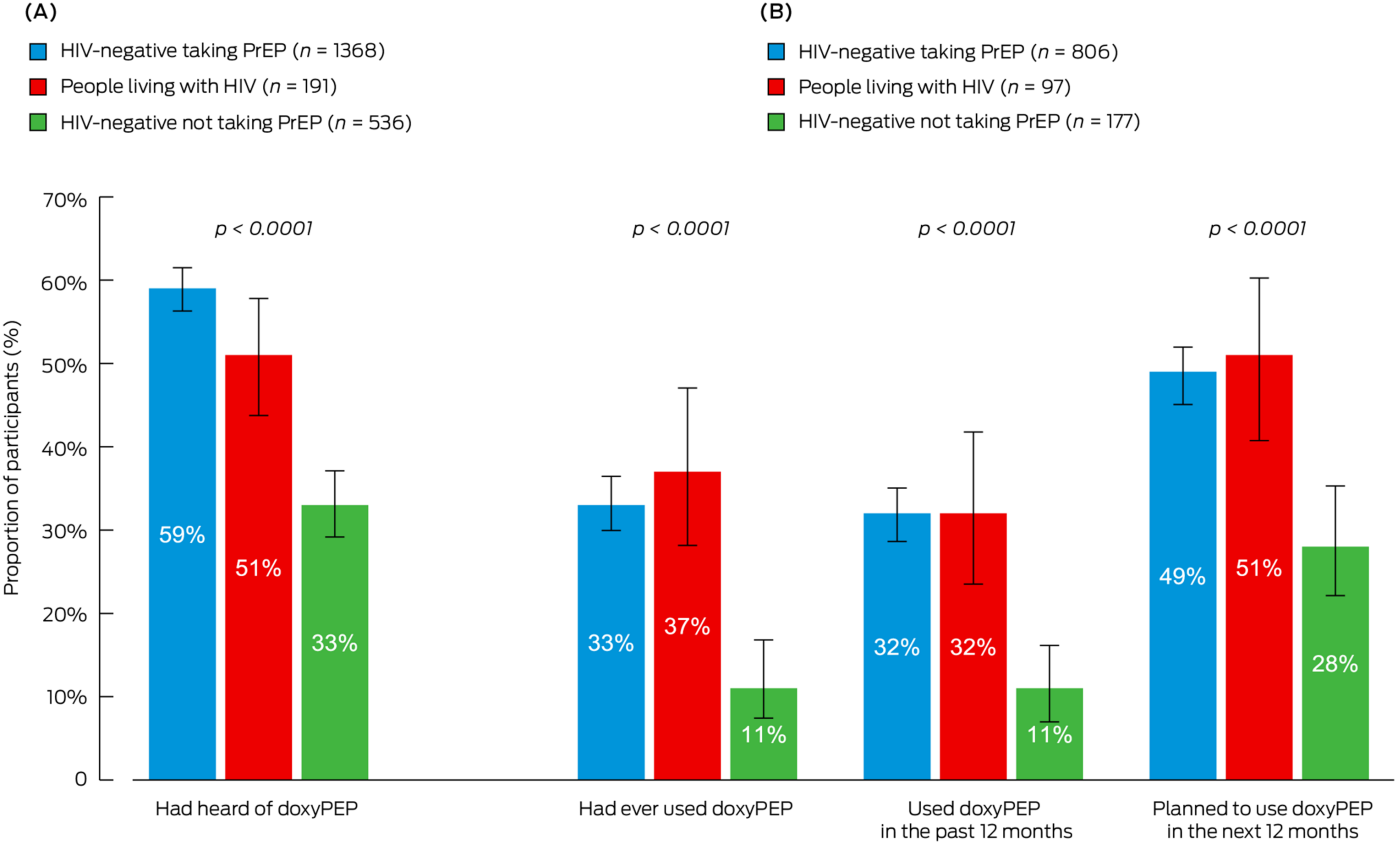
(A) Doxycycline post‐exposure prophylaxis (doxyPEP) awareness among all 2095 participants; and (B) current and future use among 1080 participants who were aware of doxyPEP, by human immunodeficiency virus (HIV) status, and pre‐exposure prophylaxis (PrEP) use. Differences in awareness and usage by HIV status and pre‐exposure prophylaxis use were statistically significant (*p* < 0.0001).

Among those aware of doxyPEP, 323/1080 (29.9%) were ever users, and 306/1080 (28.3%) were recent users. People living with HIV had the highest ever and recent doxyPEP use, followed by PrEP users and non‐PrEP users (Figure 1). Usage did not differ by recruitment setting (Table S5).

Among ever users, 205/323 (63.5%) took the recommended 200 mg within 72 h after sex, with most taking it within 24 h (121/205, 59.0%), followed by within 25–48 h (47/205, 22.9%) and 49–72 h (37/205, 18.0%). Only 4/323 (1.2%) took it every day (i.e., doxyPrEP), and the remainder 114/323 (35.3%) used other non‐recommended regimens (3/323, 0.9%).

The most common source of doxycycline was a clinician in Australia (135/323, 41.8%). However, many participants acquired doxycycline through unsupervised means, including from a friend or sexual partner (50/323, 15.5%), using leftover antibiotics from a previous treatment (47/323, 14.6%), or purchasing it online (17/323, 5.3%) or overseas (28/323, 8.7%) without a prescription (Table S6). About one‐third (105/323, 32.5%) of ever users had ever given or received doxyPEP from others. A small proportion of participants aware of doxyPEP (66/1080, 6.1%) reported using antibiotics other than doxycycline to prevent STIs (Table S7).

In the multivariable logistic regression analysis, recent doxyPEP use was associated with being a person living with HIV (aOR, 2.82; 95% CI, 1.45–5.48) or an HIV PrEP user (aOR, 2.97; 95% CI, 1.77–4.97) compared with a non‐HIV PrEP user. Other key predictors included attending a sex party in the past 12 months (aOR, 2.04; 95% CI, 1.53–2.72) and being in a sexual relationship (aOR, 1.62; 95% CI, 1.22–2.15). Among recent users, 29/306 (9.5%) reported recent syphilis diagnoses in the past 12 months, and 85/306 (27.8%) had two or more STI diagnoses in the past 12 months (Table 2).

**TABLE 2:** Factors associated with doxycycline post‐exposure prophylaxis use in the last 12 months among 1072 participants.

Characteristics	*N* (%)	OR (95% CI)	aOR (95% CI)^a^
Age (years)	—	1.01 (1.00–1.02)	—
Country of birth			
Australia/New Zealand	191/681 (28.0%)	1	—
Other	115/391 (29.4%)	1.07 (0.81–1.41)	—
Highest education			
High school	29/145 (20.0%)	1	—
Tertiary diploma or trade certificate	52/169 (30.8%)	1.78 (1.06–3.00)	—
University	225/758 (29.7%)	1.69 (1.09–2.61)	—
Type of recruitment			
Clinical	106/400 (26.5%)	1	—
Non‐clinical	200/672 (29.8%)	1.18 (0.89–1.55)	—
HIV and pre‐exposure prophylaxis use			
HIV negative, not taking pre‐exposure prophylaxis	19/176 (10.8%)	1	1
HIV negative, taking pre‐exposure prophylaxis	256/800 (32.0%)	3.89 (2.36–6.40)	2.98 (1.77–4.97)
People living with HIV	31/96 (32.3%)	3.94 (2.08–7.47)	2.82 (1.45–5.48)
Relationship status			
Single and sexually active	148/580 (25.5%)	1	1
Not sexually active	3/40 (7.5%)	0.24 (0.07–0.78)	0.53 (0.15–1.79)
In a sexual relationship	152/434 (35.0%)	1.57 (1.20–2.06)	1.62 (1.22–2.15)
Prefer not to answer or other	3/18 (16.7%)	0.58 (0.17–2.04)	0.91 (0.25–3.31)
Number of sexual partners in the past 12 months	—	1.01 (1.00–1.01)	1.00 (1.00–1.01)
Sex party or orgy in the past 12 months			
No	121/603 (20.1%)	1	1
Yes	185/469 (39.4%)	2.59 (1.98–3.41)	2.04 (1.53–2.72)
Had casual sex while travelling on a holiday			
No	72/347 (20.7%)	1	—
Yes	234/725 (32.3%)	1.82 (1.34–2.46)	—
Recent syphilis diagnosis^b^			
No	277/975 (28.4%)	1	—
Yes	29/97 (29.9%)	1.07 (0.68–1.69)	—
Two or more recent other bacterial STI diagnoses but not syphilis^b^			
No	229/832 (27.5%)	1	—
Yes	77/240 (32.1%)	1.24 (0.91–1.70)	—

Abbreviations: aOR, adjusted odds ratio; CI, confidence intervals; HIV, human immunodeficiency virus; OR, odds ratio; STI, sexually transmitted infection.

^a^ The multivariable logistic regression was built using a backward elimination approach. Variables with *p* < 0.20 in the univariable analysis (i.e., age, highest education, HIV status and pre‐exposure prophylaxis use, relationship status, number of sexual partners, sex party, casual sex while travelling and ≥ 2 recent other bacterial STIs) were included in the initial multivariable logistic regression model. The final multivariable logistic regression model presented in the table only includes variables with *p* < 0.05. The Hosmer–Lemeshow test showed that there was no evidence of lack of fit in the multivariable logistic regression model (*χ* ^2^(8) = 6.86, *p* = 0.552) and the C‐statistic of 0.679 suggested a moderate degree of model strength.

^b^ A recent diagnosis refers to a diagnosis in the past 12 months.

Among participants who were aware of doxyPEP but had not used it, 490/1080 (45.4%) planned to use it in the next 12 months, whereas 439/1080 (40.6%) were unsure.

### 3.4 Beliefs and Motivations

Most participants took doxyPEP to reduce their risk of getting STIs from their partners (269/323, 83.3%) or avoid STI treatments such as injections (201/323, 62.2%). In contrast, only 52/323 participants (16.1%) reported taking doxyPEP because a healthcare professional recommended it (Table 3). Ever users intended to prevent chlamydia (292/323, 90.4%), gonorrhoea (263/323, 81.4%) and syphilis (225/323, 69.7%), although some participants targeted infections without proven efficacy, including *Mycoplasma genitalium* (35/323, 10.8%) and herpes (26/323, 8.0%) (Figure S1 and Table S8).

**TABLE 3:** Reasons for taking doxycycline post‐exposure prophylaxis (doxyPEP) among participants who had ever used it and not taking doxyPEP among participants who had never used it.

	Number (%)
Reasons for taking doxyPEP	
Total number of participants	323
Reduce my risk of getting an STI from my partner(s)	269 (83.3%)
Reduce the need for an STI treatment, such as an injection to treat gonorrhoea or syphilis	201 (62.2%)
Reduce the risk of passing an STI to my casual sex partner(s)	177 (54.8%)
Reduce the risk of passing an STI to my regular sex partner(s)	157 (48.6%)
I think my risk of getting a STI is high	153 (47.4%)
Recommended by a doctor or health professional	52 (16.1%)
My partner did not want to use a condom	36 (11.1%)
Reduce how often I need to get tested for STIs	33 (10.2%)
Other^a^	15 (3.4%)
Reasons for not taking doxyPEP	
Total number of participants	756
Not enough information for me to decide if I should take doxyPEP	312 (41.3%)
I am worried about increasing the risk of antibiotic resistance in my community (i.e., antibiotics used to treat infections in the community are no longer effective)	256 (33.9%)
I am worried that I will develop antibiotic resistance (i.e., the antibiotics I take no longer cure infections I get)	249 (32.9%)
Other^b^	174 (23%)
I have difficulties getting a prescription	138 (18.3%)
I think my risk of getting an STI is low	133 (17.6%)
I am worried about any side effects from doxycycline	131 (17.3%)
I cannot afford the costs, including the antibiotics and the doctor's consultations	82 (10.8%)
I have difficulties getting the antibiotic	72 (9.5%)
I do not want people to know I am taking doxyPEP	31 (4.1%)
I am allergic to doxycycline or have previously had a bad reaction to doxycycline	16 (2.1%)

Abbreviation: STI, sexually transmitted infection.

^a^ Other reasons for taking doxyPEP include working in the sex industry; taking doxycycline for skin conditions but also taking it for an additional layer to protect against STI; doxycycline was offered by partners; and having sexual contact with someone who had an STI.

^b^ Other reasons for not taking doxyPEP include that they did not know it was available in Australia, did not know how to access doxyPEP, doxyPEP use eligibility, were not offered by their doctors, doctors were reluctant to prescribe doxyPEP to them, and they were embarrassed to discuss this with their general practitioner.

Of the 490 planned users, most hoped to prevent chlamydia (460/490, 93.9%), gonorrhoea (422/490, 86.1%) and syphilis (386/490, 78.8%), but some intended to prevent *M. genitalium* (92/490, 18.8%), HIV (50/490, 10.2%) and mpox (36/490, 7.4%) (Figure S1 and Table S8).

Regarding STI testing practice, the majority of ever users (292/323, 90.4%) reported that their STI testing frequency had not changed since starting doxyPEP, while 15/323 (4.6%) tested less and 14/323 (4.3%) tested more frequently.

Participants rated the community benefit of doxyPEP highly (median, 8/10; IQR, 7–10) (Table S9).

### 3.5 Concerns and Barriers

Among those who were aware of doxyPEP but had ever used it, antimicrobial resistance was the primary concern, with 170/323 (52.6%) worried about developing personal antibiotic resistance and 169/323 (52.3%) concerned about community‐level resistance. However, 129/323 (39.9%) reported no concerns about taking doxyPEP. Among those who were aware of doxyPEP but had never used it, insufficient information was the primary barrier to doxyPEP adoption (312/756, 41.3%), followed by antimicrobial resistance, specifically that antibiotics would become ineffective for the community (256/756, 33.9%) or that they would personally develop resistance to antibiotics (249/756, 32.9%) (Table 3).

Word cloud (Figure S2) and structural topic modelling analyses (Table 4) showed that participants emphasising ‘prevention’ (*β* = 0.0299; 95% CI, 0.0187–0.0411) and ‘STI reduction’ (*β* = 0.0191; 95% CI, 0.0097–0.0285) rated community benefit higher, while those emphasising ‘concerns and uncertainties’ (*β* = −0.0147; 95% CI, −0.0225 to −0.0069) and ‘misconceptions’ (*β* = −0.026; 95% CI, −0.037 to −0.0146) rated it lower (Section S1).

**TABLE 4:** Topic word profiles, relevance, interpretations and regression with perceived benefit.

Topic	Relevance	FREX Word profile	Interpretation	Interpretation keyword	*β*	95% CI
1	13.6%	Community, sex, GBMSM, infect	DoxyPEP would be relevant to GBMSM community for STIs	Community	−0.0012	−0.0094 to 0.0118
2	19.9%	Prevent, great, help, better	DoxyPEP could be great and helpful for prevention	Prevention	0.0299	0.0187 to 0.0411
3	15.3%	doxyPrEP, take, condom, drug	DoxyPEP is like another HIV PrEP that you use before/during sex, such as condoms	Misconception	−0.026	−0.037 to −0.0146
4	11.6%	Everyone, beneficial, sexual, meaningful	DoxyPEP would be sexually beneficial and meaningful for everyone	Beneficial	0.0015	−0.0081 to 0.0111
5	12.6%	Community, syphilis, long, spread	DoxyPEP would have a long impact on preventing the spread of syphilis	Syphilis control	−0.0088	−0.0188 to 0.0012
6	17.3%	Reduction, less, STI, good	DoxyPEP can effectively reduce STIs	STI reduction	0.0191	0.0097 to 0.0285
7	9.7%	Antimicrobial resistance, increase, number, unsure	Concerns and uncertainties on antimicrobial resistance due to the use of doxyPEP	Concerns and uncertainties	−0.0147	−0.0225 to −0.0069

*Note:* FREX (Frequency‐Exclusivity) words are those that are both frequent within a topic and exclusive to it, helping to identify the most distinctive words for each topic in structural topic modelling.

Abbreviations: CI, confidence interval; doxyPEP, doxycycline post‐exposure prophylaxis; doxyPrEP, doxycycline pre‐exposure prophylaxis; GBMSM, gay and bisexual men who have sex with men; HIV, human immunodeficiency virus; PrEP, pre‐exposure prophylaxis; STI, sexually transmitted infection.

## 4 Discussion

In this national survey of 2095 Australian gay and bisexual men, trans and gender diverse people, about half were aware of doxyPEP, of whom nearly one‐third had ever or recently used it. This level of awareness is similar to that reported in two recent 2024 studies in the United States [13, 15]. Recent use of doxyPEP in our study was higher than in the studies conducted in the United States [15] (14.6% vs. 13.0%) and Australia among HIV PrEP users (10.6%) [11]. Despite this uptake, doxyPEP implementation remains challenging, complicated by three critical gaps. First, a substantial proportion of use occurs outside clinical guidance, with over one‐third of users reporting non‐recommended dosage regimens and nearly half obtaining doxycycline through unsupervised channels, while a small but notable proportion reported using other, ineffective antibiotics for STI prevention. Second, user expectations exceed evidence, with most users intending to prevent gonorrhoea, despite the data showing limited efficacy. Third, uptake was driven more by situational risk factors, such as attending sex parties, than by a recent STI history as recommended in the guidelines. These findings highlight the need for educational strategies to ensure safe and effective use, while managing risks.

Non‐recommended regimens and informal sourcing point to a disconnect between community adoption and clinical guidance. Users are improvising with daily doxyPrEP‐style use or 2‐1‐1 schedules (i.e., two doses before sexual exposure, followed by one dose at 24 h and one at 48 h after the first dose), suggesting adaptation of familiar HIV prevention strategies. This practice is likely driven by both reliance on informal channels, such as friends, and the potential complexity of managing different on‐demand regimens for HIV PrEP and STIs, which contrasts sharply with the participants' stated trust in healthcare professionals. With nearly one in seven users obtaining doxyPEP without prescriptions, rapid community uptake is outpacing clinical access. If prescribing practices remain restrictive, unsupervised use will only increase as demand continues to grow. Sourcing medication through these unsupervised channels creates tangible risks, including the potential for adverse effects [25] such as pill‐induced oesophagitis or allergic reactions.

Our analysis found that community usage patterns align with some aspects of the Australian statement while diverging from others. Although the statement recommends doxyPEP primarily for individuals with recent syphilis diagnoses or multiple bacterial STIs [4], we found no association between these reported clinical indicators and actual use. Instead, the strongest predictors were situational risk factors, such as attending sex parties. Notably, the statement does recognise situational risk assessment, specifically recommending doxyPEP for ‘GBMSM who identify an upcoming period of heightened STI risk [for example], attendance at a sex event’ [4]. This pattern contrasts with US findings, where recent STI or syphilis diagnoses were significantly associated with use [15], suggesting that Australian users may be implementing the situational risk guidance more readily than the clinical history criteria. This prospective, risk‐based approach represents rational prevention decision‐making, in which individuals assess their own risk before infection occurs rather than waiting for a diagnosis. Such self‐assessment should be recognised as a valid basis for doxyPEP initiation. This is further complicated by user motivations that conflict with clinical guidance, including a desire to reduce STI testing frequency or to avoid recommended injectable treatments for STIs. Such preferences may reflect the community's appetite for reducing asymptomatic screening for chlamydia and gonorrhoea to reduce antimicrobial resistance, a topic of growing debate in sexual health [16, 26, 27].

Our findings reveal concerning misconceptions about doxyPEP. Despite meta‐analyses demonstrating only 22% effectiveness against gonorrhoea [5], 81.4% believed doxyPEP would prevent it. Australia has high baseline tetracycline resistance rates (31% [28] to 62% [29] vs. 20%–35% in doxyPEP trial settings [30]), further compromising its effectiveness, yet this important context appears absent from community knowledge. Some participants intended to use doxyPEP to prevent HIV (10.2%) and mpox (7.4%). These knowledge deficits are exacerbated by information barriers, with 41.3% of non‐users citing insufficient information as their primary concern. This challenge is compounded by substandard online resources and online testing options, as evidenced by recent reviews showing that most Australian STI testing websites fail to meet national management guidelines [31]. These findings underscore the need for evidence‐based educational resources that accurately convey both benefits and limitations, addressing widespread misconceptions.

First, educational materials must be co‐developed with clinicians and community organisations to ensure that the information is clear and direct and effectively reaches the community [32]. This education needs to manage expectations by accurately stating the limited effectiveness of doxyPEP against gonorrhoea in the Australian context and clarifying what it does not prevent, including HIV or mpox. Second, policies must improve clinical access to doxyPEP to reduce unsupervised use while increasing oversight of online providers; a national quality framework, similar to the one in development for STI testing services, could provide a useful model. Finally, future research should focus on how to improve communication strategies, tailoring eligibility criteria and optimising implementation while preserving antimicrobial stewardship.

Our study was a large national co‐design survey with dual recruitment (clinical and community settings), inclusion of trans and gender diverse people, and structural topic modelling of qualitative data.

### 4.1 Limitations

Our study has several limitations. First, as the survey participants were self‐selecting, non‐probabilistic sample, the generalisability of the survey findings cannot be assessed. Second, the cross‐sectional design allows us to identify associations but not to establish causality between risk factors and doxyPEP use. Additionally, we used backward elimination for variable selection in our multivariable model, which has known limitations, including potential model instability. Future research could employ alternative approaches, such as Akaike Information Criterion/Bayesian Information Criterion (AIC/BIC)‐based selection or cross‐validation, to validate our findings. Third, although we recruited from diverse settings, particularly those not engaged with sexual health services or online platforms, findings are not generalisable to people in heterosexual relationships or female sex workers, who may also use doxyPEP. In addition, although we used multiple methods to detect and prevent duplicate entries, including IP address matching, some flagged duplicates may represent legitimate participants using shared networks at clinical sites or community organisations rather than true duplicates. Fourth, self‐reported data on doxyPEP usage, sexual practices and STI diagnoses may be subject to recall bias and social desirability bias. Fifth, this survey captured community perspectives and experiences that may differ from clinicians' intentions or explanations provided during consultations. Clinical practice patterns and prescribing decisions are examined in a separate study. Finally, our findings reflect implementation patterns during the early rollout phase and may not represent longer‐term usage dynamics as doxyPEP becomes more established in clinical practice.

## 5 Conclusions

Although doxyPEP use was common, implementation is complex. Community practice is largely driven by situational risk assessments and informal channels, leading to non‐recommended regimens and unsupervised sourcing. Substantial barriers persist, including insufficient information and limited clinical access. Therefore, as doxyPEP implementation expands, an urgent coordinated action is needed between health departments and community organisations to deliver evidence‐based education and improve access, ensuring safe and effective use.

## Author Contributions

Eric P.F. Chow was involved in the conceptualisation of the study, oversaw the study and secured funding for this study. Eric P.F. Chow and Fabian Y.S. Kong developed the methodology. Phyu Mon Latt and Eric P.F. Chow performed the data analyses and wrote the first draft of the manuscript. Eric P.F. Chow, Fabian Y.S. Kong, Dash Heath‐Paynter, Vincent J. Cornelisse, Michael W. Traeger, Daniel Grace and Kai J. Jonas developed the study materials. Phyu Mon Latt prepared the visualisations. Kate Maddaford, Teralynn Ludwick, Ethan T. Cardwell and Fabian Y.S. Kong conducted pre‐testing of the survey. Eric P.F. Chow and Haoyi Wang conducted the text cleaning and word cloud visualisation of participants' free‐text responses using text mining methods in R. Haoyi Wang performed topic modelling to explore key themes from free‐text responses. Kate Maddaford coordinated the preparation and distribution of study materials across clinical sites. Eric P.F. Chow, Ei Thu Aung, Kate Maddaford, Christopher K. Fairley, Sarah J. Martin, Carole Khaw, Rick Varma, Caroline Thng, Manoji Gunathilake, Vincent J. Cornelisse, Dash Heath‐Paynter and Benjamin Riley were involved in study recruitment and data collection. All authors revised the manuscript critically for important intellectual content and approved the final version of the manuscript.

## Funding

This study was supported by Eric P.F. Chow's Australian National Health and Medical Research Council (NHMRC) Investigator Grant (GNT1172873). Eric P.F. Chow, Christopher K. Fairley, Catriona S. Bradshaw and Fabian Y.S. Kong are each supported by an Australian NHMRC Investigator Grant (GNT2033299, GNT1172900, GNT1173361 and GNT2033078, respectively). Ei Thu Aung and Phyu Mon Latt are supported by Eric P.F. Chow's NHMRC Investigator Grant (GNT2033299). Daniel Grace is supported by a Canada Research Chair in Sexual and Gender Minority Health. Caroline Thng provided in‐kind support within her role at Gold Coast Hospital and Health Service. The funders of the study had no role in the study design, data collection, data analysis, data interpretation or the writing of this report.

## Disclosure

Not commissioned; externally peer reviewed.

## Conflicts of Interest

The authors declare no conflicts of interest.

## Supporting information


**Data S1:** mja270180‐sup‐0001‐Supinfo.pdf.

## Data Availability

The data supporting the findings of this study are available within the article and its Supporting Information. Data will be shared upon reasonable request to the corresponding author with permission from the Alfred Hospital Ethics Committee.
