# Supplementary material for: Awareness, Usage and Perceptions of Doxycycline Post‐Exposure Prophylaxis (doxyPEP) for Prevention of Sexually Transmitted Infections in Australia: Insights From a National Cross‐Sectional Survey
Source: Med J Aust. 2026 Apr 14;224(4):e70180. doi: 10.5694/mja2.70180 (PMC13077732; doi:10.5694/mja2.70180)
Supplement: Supplementary file 1 — Data S1: mja270180‐sup‐0001‐Supinfo.pdf. [file MJA2-224-0-s001.pdf]

Supporting Information

Supplementary material

This appendix was part of the submitted manuscript and has been peer reviewed. It is posted as supplied by the authors.

Appendix to: P. M. Latt, E. T. Aung, K. Maddaford, et al. Awareness, Usage And Perceptions of Doxycycline Post-Exposure Prophylaxis (doxyPEP) for Prevention of Sexually Transmitted Infections in Australia: Insights From a National Cross-Sectional Survey. *Medical Journal of Australia* 2026; doi: 10.5694/mja2.70180.

Contents

Table S1. Participant recruitment by site and channel 3

Table S2. Survey participant flow and exclusion reasons 4

Table S3. Ever heard of doxycycline post-exposure prophylaxis among 2095 participants. 4

Table S4. Weighted mean trust scores for doxycycline post-exposure prophylaxis information sources among 1080 participants who were aware of doxycycline post-exposure prophylaxis. 4

Table S5. Ever and recent doxycycline post-exposure prophylaxis use among 1080 participants who were aware of doxycycline post-exposure prophylaxis, stratified by type of recruitment. 5

Table S6. Sources of obtaining doxycycline post-exposure prophylaxis among 323 ever doxycycline post-exposure prophylaxis users. 5

Table S7. List of antibiotics used by 66 participants who reported using antibiotics other than doxycycline. 6

Table S8. Infections that participants hoped to prevent with doxycycline post-exposure prophylaxis 6

Table S9. Score on the benefit of doxycycline post-exposure prophylaxis to the community among 1080 participants who were aware of doxycycline post-exposure prophylaxis. 7

Figure S1. Infections participants hoped to prevent with doxycycline post-exposure prophylaxis among 323 ever users of doxycycline post-exposure prophylaxis, and 490 planned to take doxycycline post-exposure prophylaxis in the next 12 months 7

Figure S2. Word clouds illustrating participants' free-text responses regarding the perceived community benefit of doxycycline post-exposure prophylaxis. Participants were asked to rate the perceived benefit of doxycycline post-exposure prophylaxis for their community on a scale from 0 (no benefit) to 10 (maximum benefit), and to describe the factors that influenced their rating. 8

Section S1 9

Qualitative Analysis of Perceived Community Benefit of Doxycycline Post-Exposure Prophylaxis 9

Checklist for Reporting of Survey Studies (CROSS) 11

DoxyAWARE Questionnaire 14

Table S1. Participant recruitment by site and channel

Recruitment Source	Number of Participants	Percentage
**Clinical Sites (Total)**	**668**	**31.89%**
Melbourne Sexual Health Centre	312	14.89%
Canberra Sexual Health Centre	101	4.82%
Adelaide Sexual Health Centre	83	3.96%
M Clinic	34	1.62%
RAPID Clinics	20	0.95%
South Terrace Clinic	17	0.81%
Sydney Sexual Health Centre	16	0.76%
Gold Coast Sexual Health Centre	14	0.67%
Prahran Market Clinic	12	0.57%
Clinic 34	10	0.48%
North Coast NSW Clinic	10	0.48%
aTest	8	0.38%
Centre Clinic	8	0.38%
Metro North Health	6	0.29%
Other clinical sites	5	0.24%
View Street Medical	4	0.19%
Ochre Medical Centre	3	0.14%
Clinic 16 – St Leonards	3	0.14%
South Melbourne Doctors	1	0.05%
East Canberra General Practice	1	0.05%
**Non-Clinical Channels (Total)**	**1427**	**68.11%**
Grindr	670	31.98%
Jackd	571	27.26%
Social media	95	4.53%
ACON/Thorne Harbour Health	26	1.24%
Friends/word-of-mouth	23	1.10%
Scruff	22	1.05%
University of Melbourne Student Portal	17	0.81%
Merdian	2	0.10%
ASHM	1	0.05%

Percentages calculated from the total sample (2095 participants). Clinical sites include sexual health clinics and general practices. Non-clinical channels include gay dating apps, social media platforms, community organisations, and a university portal.

Table S2. Survey participant flow and exclusion reasons

Category	Number
**Total individuals who initiated the survey**	**3,940**
**Excluded participants**	**1,845**
Did not meet eligibility criteria	1,239
Did not submit the survey	320
Duplicate entries (same IP address)	247
Poor-quality responses	29
Did not provide consent	10
**Final sample for analysis**	**2,095**

Eligibility criteria included: (1) aged ≥18 years; (2) identified as a man (cis or trans), trans woman, or non-binary person who has sex with men; (3) currently living in Australia; and (4) Year 10 level English proficiency. Poor-quality responses were identified through attention check questions. Duplicate entries were identified through IP address matching.

Table S3. Ever heard of doxycycline post-exposure prophylaxis among 2095 participants.

Ever heard of doxycycline post-exposure prophylaxis	Clinical settings	Non-clinical settings	P
Participants	668	1427	
Yes	403 (60.3%)	677 (47.4%)	<0.0001
No	243 (36.4%)	696 (48.8%)	
I do not know	22 (3.3%)	54 (3.8%)	

Table S4. Weighted mean trust scores for doxycycline post-exposure prophylaxis information sources among 1080 participants who were aware of doxycycline post-exposure prophylaxis.

Sources	Rank 1	Rank 2	Rank 3	Total score by source†	Weighted mean score‡
Healthcare professionals	909	107	33	2974	2.8
Peer community organisations	84	499	155	1405	1.3
Online providers of doxycycline post-exposure prophylaxis	17	176	201	604	0.6
Friends	16	104	237	493	0.5
Media	3	49	154	261	0.2
Sex partners	13	40	91	210	0.2
Online community group or forum	3	40	109	198	0.2
Other*	24	22	31	147	0.1

**Other sources include government, and professional health organisations (e.g. Centers for Disease Control and Prevention (CDC), ASHM Health)*

† Rank 1 was assigned with 3 points, rank 2 was assigned with 2 points and rank 3 was assigned with 1 point. The total score by source was the sum of the points in each source.
‡ The weighted mean score for each source was calculated by dividing its total score by the total number of participants.

Table S5. Ever and recent doxycycline post-exposure prophylaxis use among 1080 participants who were aware of doxycycline post-exposure prophylaxis, stratified by type of recruitment.

	**Clinical settings**	**Non-clinical settings**	**P**
Participants	403	677	0.301
Ever doxycycline post-exposure prophylaxis users			0.301
Yes	113 (28.0%)	210 (31.0%)	
No	290 (72.0%)	467 (69.0%)	
Recent doxycycline post-exposure prophylaxis users			0.253
Yes	106 (26.3%)	200 (29.5%)	
No	297 (73.7%)	477 (70.5%)	

Table S6. Sources of obtaining doxycycline post-exposure prophylaxis among 323 ever doxycycline post-exposure prophylaxis users.

**Sources***	**Number (%)**
Directly from a general practitioner/doctor in Australia (in-person or telehealth consult)	135 (41.8%)
From a local pharmacy in Australia with a prescription	122 (37.8%)
Online with a prescription†	15 (4.6%)
Online without a prescription‡	17 (5.3%)
In-person from another country with a prescription§	19 (5.9%)
Argentina	*1 (6.7%)*
Brazil	*2 (13.3%)*
Canada	*2 (13.3%)*
China	*2 (13.3%)*
Germany	*1 (6.7%)*
New Zealand	*3 (20.0%)*
United Kingdom	*1 (6.7%)*
United States	*1 (6.7%)*
Taiwan	*1 (6.7%)*
Thailand	*1 (6.7%)*
In-person from another country without a prescription¶	28 (8.7%)
Argentina	1 (4.5%)
Cambodia	*2 (9.1%)*
China	*1 (4.5%)*
India	*1 (4.5%)*

Indonesia	*3 (13.6%)*
Mexico	*3 (13.6%)*
Qatar	*1 (4.5%)*
United States	*2 (9.1%)*
Thailand	*10 (45.5%)*
Vietnam	*1 (4.5%)*
From a friend or sex partner	50 (15.5%)
From a trial or research study	5 (1.5%)
Left over from another treatment	47 (14.6%)
Other‖	7 (2.2%)

** Participants can select more than one source of obtaining doxyPEP.*
† Participants reported obtaining doxycycline online without a prescription, including AllDayChemist.com and Hey Fella.
‡ Participants reported obtaining doxycycline online with a prescription, including Doctors On Demand, Healthylife Pharmacy, InstantScripts and Hey Fella.
§ Of the 19 participants, 15 reported the country where they obtained doxyPEP with a prescription, and therefore we used 15 as the denominator to calculate the proportion of each country.
¶ Of the 28 participants, 22 reported the country where they obtained doxyPEP without a prescription, and 3 reported two countries. Therefore, we used 22 as the denominator to calculate the proportion of each country.
‖ Seven participants also reported other sources of obtaining doxyPEP, including obtaining doxycycline for other purposes (e.g. acne and malaria prevention) and decided to use the remaining doxycycline as doxyPEP for STI prevention.

Table S7. List of antibiotics used by 66 participants who reported using antibiotics other than doxycycline.

**Antibiotic**	**Number (%)**
Amoxicillin	20 (30.3%)
Azithromycin	33 (50.0%)
Ceftriaxone	11 (16.7%)
Ciprofloxacin	7 (10.6%)
Erythromycin	3 (4.5%)
Penicillin	12 (18.2%)

Table S8. Infections that participants hoped to prevent with doxycycline post-exposure prophylaxis.

**Infections**	**Participants who had ever taken doxycycline post-exposure prophylaxis**	**Participants who planned to take doxycycline post-exposure prophylaxis in the next 12 months**
Participants	323	490
Chlamydia	292 (90.4%)	460 (93.9%)
Gonorrhoea	263 (81.4%)	422 (86.1%)
Genital warts	20 (6.2%)	72 (14.7%)
Herpes	26 (8.0%)	90 (18.4%)
HIV	23 (7.1%)	50 (10.2%)
Mpox	10 (3.1%)	36 (7.4%)
Mycoplasma genitalium	35 (10.8%)	92 (18.8%)
Syphilis	225 (69.7%)	386 (78.8%)

Table S9. Score on the benefit of doxycycline post-exposure prophylaxis to the community among 1080 participants who were aware of doxycycline post-exposure prophylaxis.

**Benefit score**	**Number (%)**
0 (no benefit)	2 (0.2%)
1	2 (0.2%)
2	8 (0.7%)
3	12 (1.1%)
4	11 (1.0%)
5	71 (6.6%)
6	57 (5.3%)
7	164 (15.2%)
8	293 (27.1%)
9	131 (12.1%)
10 (maximum benefit)	329 (30.5%)

Figure S1. Infections participants hoped to prevent with doxycycline post-exposure prophylaxis among 323 ever users of doxycycline post-exposure prophylaxis, and 490 planned to take doxycycline post-exposure prophylaxis in the next 12 months

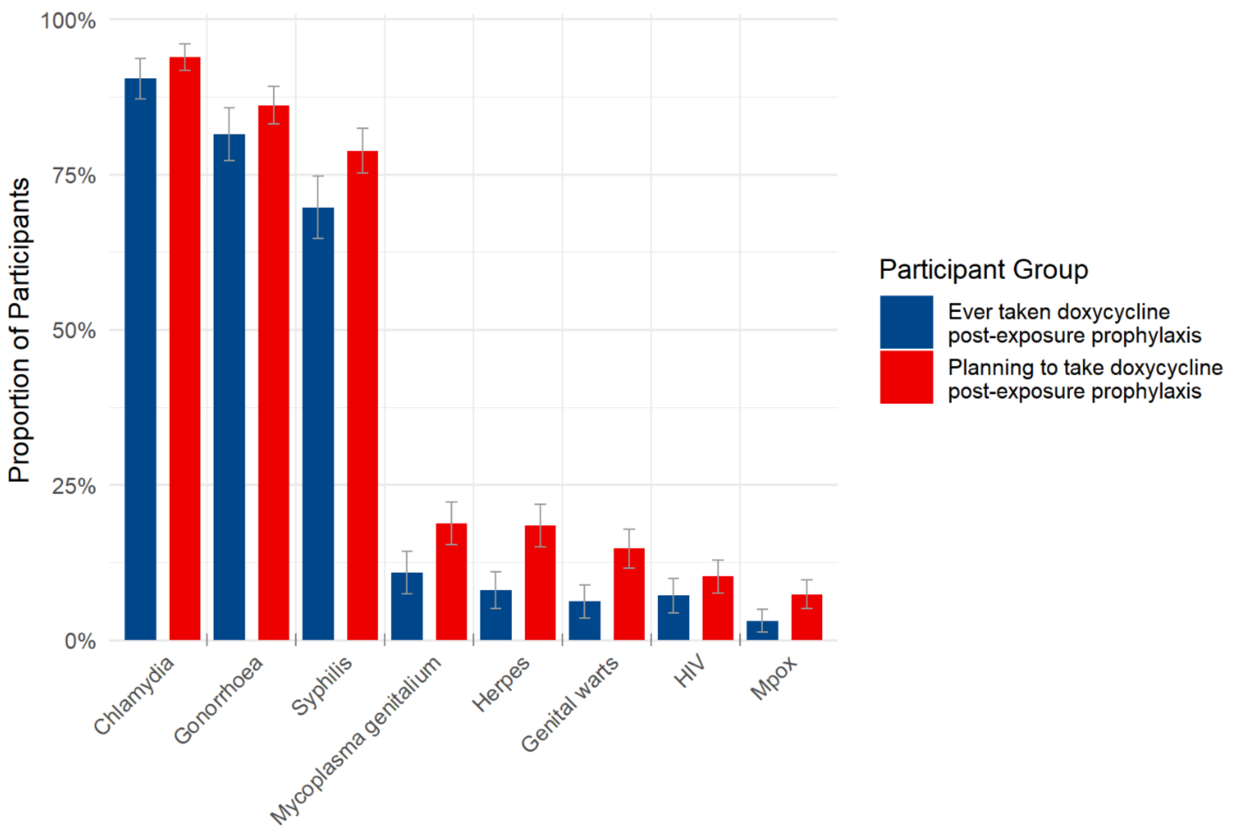

Figure S2. Word clouds illustrating participants' free-text responses regarding the perceived community benefit of doxycycline post-exposure prophylaxis. Participants were asked to rate the perceived benefit of doxycycline post-exposure prophylaxis for their community on a scale from 0 (no benefit) to 10 (maximum benefit), and to describe the factors that influenced their rating.

A. All participants (1080); the five most frequent words were STI (341), community (135), risks (99), reduce (98), and people (76).
B. Participants with high scores (6-10, 974); the five most frequent words were STI (314), community (115), reduce (94), risks (80), and people (69).
C. Participants with low scores (0-5, 106); the top five words were antibiotics (31), antimicrobial resistance (29), STI (27), community (20), and risks (19).

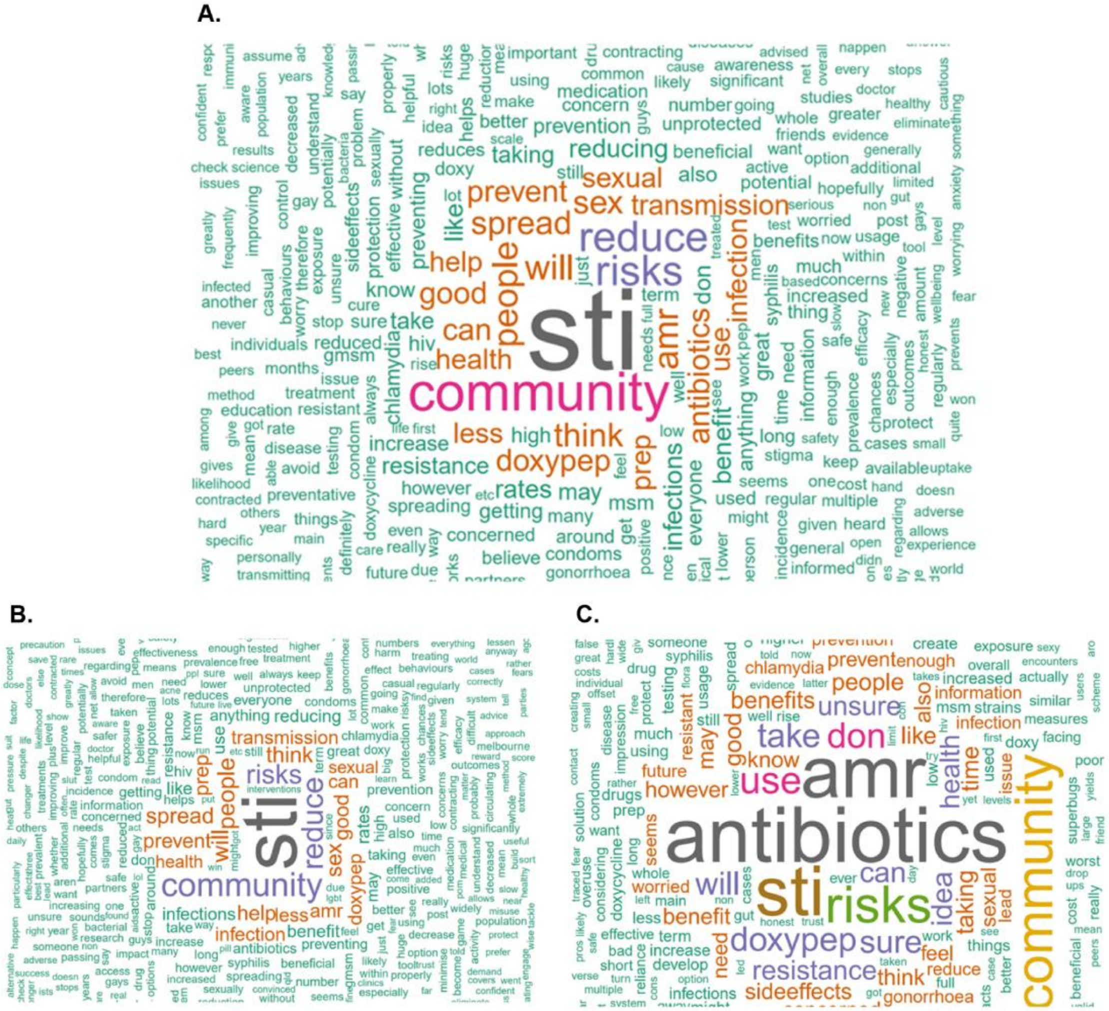

Section S1

Qualitative Analysis of Perceived Community Benefit of Doxycycline Post-Exposure Prophylaxis

Overview of Analytical Approach

We analysed open-ended text responses about the perceived community benefits and harms of doxycycline post-exposure prophylaxis (doxyPEP) using two complementary approaches: word cloud visualisation and structural topic modelling (STM). While word clouds provide an accessible visual summary of frequently mentioned terms, STM offers a more sophisticated analytical framework by identifying latent thematic structures and quantifying their associations with participants' numerical benefit ratings.

Word Cloud Analysis

Word clouds were generated using the “wordcloud” package in R to visualise the most frequently occurring terms in participants' responses. Figure S2 presents three word clouds:

- Panel A (All participants, 1080): The five most frequent terms were STI (341), community (135), risks (99), reduce (98), and people (76).
- Panel B (High perceived benefit scores 6-10, 974): The five most frequent terms were STI (314), community (115), reduce (94), risks (80), and people (69).
- Panel C (Low perceived benefit scores 0-5, 106): The five most frequent terms were antibiotics (31), antimicrobial resistance (AMR) (29), STI (27), community (20), and risks (19).

The word clouds illustrate that participants with high benefit scores emphasised STI prevention and risk reduction, while those with low scores focused on antibiotic-related concerns and antimicrobial resistance.

Structural Topic Modelling Methodology

Structural topic modelling was used to identify latent topics from participants' free-text responses. STM, as an unsupervised machine learning technique, allows topic estimation while incorporating document-level covariates and is well-suited for short, heterogeneous responses typical of survey-based qualitative data, making it uniquely suited to social science and public health research. Prior to STM, all open-ended responses were pre-processed using an iterative text normalisation pipeline by removing punctuation and numbers, converting all words into lowercase, and removing stopwords. This text normalisation step was conducted by HW and EC, with inter-coder agreement established.

The optimal number of topics (seven) was determined using model diagnostics, including held-out likelihood, residual analysis, semantic coherence, and lower bound score. For each topic, we extracted the most relevant and exclusive words using FREX (frequency and exclusivity) scores, which balance word proportion within the topic against exclusivity to that topic. We considered models with higher held-out likelihood, lower residuals, and greater semantic coherence to be better-fitting, and those with higher lower bound values to exhibit more robust convergence. Based on these criteria, we selected the model that demonstrated the best balance of performance and convergence across all metrics.

We then examined the association between each topic's posterior probability in a participant's response and their perceived benefit score (rated 0-10) using linear regression. The regression coefficients (β) quantify the relationship between topic emphasis and benefit ratings. For the structural topic model, we reported regression coefficients (β), which represented the estimated change in perceived benefit score associated with each topic. Positive β values indicate topics associated with higher perceived benefit, while negative values indicate lower perceived benefit.

Interpreting Regression Coefficients (β)

The β coefficient represents the estimated change in perceived benefit score (on a 0-10 scale) associated with a one-unit increase in topic prevalence.

- Positive β values indicate that emphasising this topic is associated with higher perceived community benefit ratings
- Negative β values indicate that emphasising this topic is associated with lower perceived community benefit ratings
- The magnitude indicates the strength of association

For example, β=0.0299 for the "prevention" topic means that participants whose responses strongly emphasised prevention themes rated doxyPEP's community benefit approximately 0.03 points higher per unit increase in topic probability, compared to those who did not emphasise this theme.

Example of detailed interpretation for Topic 6 (STI reduction) [Relevance: 17.3%]

FREX words: reduction, less, STI, good

Interpretation: Responses emphasising that doxyPEP can effectively reduce sexually transmitted infections overall.

Association with perceived benefit: β = 0.0191 (95% CI: 0.0097 to 0.0285), $P < 0.001$

Interpretation: Strongly positive association. Participants who emphasised overall STI reduction (rather than specific infections) rated community benefit significantly higher. This suggests that understanding doxyPEP's broad protective effects is associated with more positive perceptions.

Please see Table 4 (main manuscript) for the complete topic modelling results for all seven topics, including FREX word profiles, relevance percentages, interpretations, and regression coefficients with 95% confidence intervals. The interpretation approach illustrated above for Topic 6 applies to all topics in Table 4.

Checklist for Reporting of Survey Studies (CROSS)

Section/topic	Item	Item description	Reported on page #
**Title and abstract**			
Title and abstract	1a	State the word "survey" along with a commonly used term in title or abstract to introduce the study's design.	1
	1b	Provide an informative summary in the abstract, covering background, objectives, methods, findings/results, interpretation/discussion, and conclusions.	1
**Introduction**			
Background	2	Provide a background about the rationale of study, what has been previously done, and why this survey is needed.	3
Purpose/aim	3	Identify specific purposes, aims, goals, or objectives of the study.	3
**Methods**			
Study design	4	Specify the study design in the methods section with a commonly used term (e.g., cross-sectional or longitudinal).	4
Data collection methods	5a	Describe the questionnaire (e.g., number of sections, number of questions, number and names of instruments used).	4
	5b	Describe all questionnaire instruments that were used in the survey to measure particular concepts. Report target population, reported validity and reliability information, scoring/classification procedure, and reference links (if any).	4 (partially applicable)
	5c	Provide information on pretesting of the questionnaire, if performed (in the article or in an online supplement). Report the method of pretesting, number of times questionnaire was pre-tested, number and demographics of participants used for pretesting, and the level of similarity of demographics between pre-testing participants and sample population.	NA
	5d	Questionnaire if possible, should be fully provided (in the article, or as appendices or as an online supplement).	Added as Supporting information Part 1
Sample characteristics	6a	Describe the study population (i.e., background, locations, eligibility criteria for participant inclusion in survey, exclusion criteria).	4
	6b	Describe the sampling techniques used (e.g., single stage or multistage sampling, simple random sampling, stratified sampling, cluster sampling, convenience sampling). Specify the locations of sample participants whenever clustered sampling was applied.	NA
	6c	Provide information on sample size, along with details of sample size calculation.	4

	6d	Describe how representative the sample is of the study population (or target population if possible), particularly for population-based surveys.	4, 9
Survey administration	7a	Provide information on modes of questionnaire administration, including the type and number of contacts, the location where the survey was conducted (e.g., outpatient room or by use of online tools, such as SurveyMonkey).	4
	7b	Provide information of survey's time frame, such as periods of recruitment, exposure, and follow-up days.	4
	7c	Provide information on the entry process:<br>–>For non-web-based surveys, provide approaches to minimize human error in data entry.<br>–>For web-based surveys, provide approaches to prevent "multiple participation" of participants.	4
Study preparation	8	Describe any preparation process before conducting the survey (e.g., interviewers' training process, advertising the survey).	4
Ethical considerations	9a	Provide information on ethical approval for the survey if obtained, including informed consent, institutional review board [IRB] approval, Helsinki declaration, and good clinical practice [GCP] declaration (as appropriate).	5
	9b	Provide information about survey anonymity and confidentiality and describe what mechanisms were used to protect unauthorized access.	4
Statistical analysis	10a	Describe statistical methods and analytical approach. Report the statistical software that was used for data analysis.	4
	10b	Report any modification of variables used in the analysis, along with reference (if available).	4
	10c	Report details about how missing data was handled. Include rate of missing items, missing data mechanism (i.e., missing completely at random [MCAR], missing at random [MAR] or missing not at random [MNAR]) and methods used to deal with missing data (e.g., multiple imputation).	4
	10d	State how non-response error was addressed.	4,8
	10e	For longitudinal surveys, state how loss to follow-up was addressed.	NA
	10f	Indicate whether any methods such as weighting of items or propensity scores have been used to adjust for non-representativeness of the sample.	NA
	10g	Describe any sensitivity analysis conducted.	NA
**Results**			
Respondent characteristics	11a	Report numbers of individuals at each stage of the study. Consider using a flow diagram, if possible.	6
	11b	Provide reasons for non-participation at each stage, if possible.	6

	11c	Report response rate, present the definition of response rate or the formula used to calculate response rate.	NA
	11d	Provide information to define how unique visitors are determined. Report number of unique visitors along with relevant proportions (e.g., view proportion, participation proportion, completion proportion).	NA
Descriptive results	12	Provide characteristics of study participants, as well as information on potential confounders and assessed outcomes.	6 (Table 1)
	13a	Give unadjusted estimates and, if applicable, confounder-adjusted estimates along with 95% confidence intervals and p-values.	6 (Table 2)
Main findings	13b	For multivariable analysis, provide information on the model building process, model fit statistics, and model assumptions (as appropriate).	Table 2 Footnote
	13c	Provide details about any sensitivity analysis performed. If there are considerable amount of missing data, report sensitivity analyses comparing the results of complete cases with that of the imputed dataset (if possible).	NA
**Discussion**			
Limitations	14	Discuss the limitations of the study, considering sources of potential biases and imprecisions, such as non-representativeness of sample, study design, important uncontrolled confounders.	8
Interpretations	15	Give a cautious overall interpretation of results, based on potential biases and imprecisions and suggest areas for future research.	8
Generalizability	16	Discuss the external validity of the results.	8
**Other sections**			
Role of funding source	17	State whether any funding organization has had any roles in the survey's design, implementation, and analysis.	Yes
Conflict of interest	18	Declare any potential conflict of interest.	Yes
Acknowledgements	19	Provide names of organizations/persons that are acknowledged along with their contribution to the research.	Yes

DoxyAWARE Questionnaire

Start of Block: Front Page. Consent

This survey is being run by the Melbourne Sexual Health Centre (MSHC) and Monash University in collaboration with researchers from Sydney Sexual Health Centre, Gold Coast Sexual Health Service, a[test], Adelaide Sexual Health Centre, Canberra Sexual Health Centre, M Clinic, Clinic 34, North Coast Sexual Health and HIV Services, UNSW Kirby Institute, The University of Melbourne, Burnet Institute, Maastricht University (the Netherlands) and The University of Toronto (Canada); and representatives from ASHM Health (the peak body representing healthcare professionals in HIV, sexual and reproductive health) and Health Equity Matters (the national peak body for the HIV community-controlled response). We would like to invite you to participate in this short survey about your attitudes towards using an antibiotic called doxycycline after sex to reduce your risk of getting sexually transmitted infections (STIs) - this is also known as doxycycline post-exposure prophylaxis (or Doxy-PEP). We would also like to understand your experience and/or views on using Doxy-PEP. Participation in this survey is voluntary and anonymous. No personally identifiable information will be collected in this survey. Your responses to this survey will remain confidential to the research team. This study has been approved by the Alfred Hospital Ethics Committee (Project 107/24). This project has also been reviewed and approved by the Human Research Ethics Committee of Northern Territory Health and Menzies School of Health Research (2024-4878) and LGBTIQA+ community health organisations including ACON (202405) and Thorne Harbour Health (THH_2024_010). A detailed Participant Information Sheet can be downloaded here. This survey will take about 5-10 minutes to complete. Upon completion of the survey, you will have the option to enter a prize draw to win one of the five $200 Visa gift cards. Please don't complete the survey more than once. This survey asks explicit questions about sexual practices. You do not have to answer questions you do not want to, and you can exit the survey at any time. If you agree to participate in this survey, please click on the "Agree" button below. If do not want to participate in this survey, please click on the "Disagree" button below.

○ Agree

○ Disagree

End of Block: Front Page. Consent

Start of Block: Bot check

Before you proceed to the survey, please click below

End of Block: Bot check

Start of Block: Block 1

To be eligible to participate in this survey, you must confirm that you meet the following inclusion criteria. *(Please select all that apply)*

- ▢ I am currently living in Australia
- ▢ I am aged 18 years old or older
- ▢ I am a man (cis or trans man), trans woman, or non-binary person who has sex with men
- ▢ I am at least at a Year 10 level in English (can read most newspaper articles)

JS

Who is the hottest man on Earth?

____

End of Block: Block 1

Start of Block: Block 2

Section A. Demographics. ***In this section, we will ask you about some basic characteristics such as age, gender, and country of birth.***

---

*

How old are you? (in years)

____

How do you describe your gender?

- ○ Man or male
- ○ Woman or female
- ○ Non-binary
- ○ Prefer not to answer
- ○ I use a different term (please specify)

____

What was your sex recorded at birth?

- ○ Male
- ○ Female
- ○ Intersex
- ○ Prefer not to answer

Do you consider yourself to be:

○ Gay or homosexual

○ Straight or heterosexual

○ Bisexual

○ Queer

○ Prefer not to answer

○ I use a different term (please specify)

____

End of Block: Block 2

Start of Block: Block 34

What is the highest level of education you have completed?

○ Year 11 or below

○ Year 12 / HSC / QCE / SACE / VCE / WACE

○ Tertiary diploma or trade certificate / TAFE

○ University degree

○ Other (please specify) ____

End of Block: Block 34

Start of Block: Block 3

What country were you born in?

▼ Afghanistan ... Prefer not to answer

End of Block: Block 3

Start of Block: Block 4

*

How many years have you been in Australia? *(Please enter a number)*

____

End of Block: Block 4

Start of Block: Block 5

In which state or territory do you currently live?

- ○ Australian Capital Territory
- ○ New South Wales
- ○ Northern Territory
- ○ Queensland
- ○ South Australia
- ○ Tasmania
- ○ Victoria
- ○ Western Australia
- ○ Other Territories
- ○ Outside Australia

End of Block: Block 5

Start of Block: Block 6

What is your current status of living in Australia?

- Australian Citizen
- Permanent Resident
- Temporary visa holder e.g., student, work
- Other (please specify) ____

End of Block: Block 6

Start of Block: Block 36

Do you have a Medicare card?

- No
- Yes
- I do not know

End of Block: Block 36

Start of Block: Block 35

Have you ever been diagnosed with HIV?

- ◯ No
- ◯ Yes
- ◯ I do not know

End of Block: Block 35

Start of Block: Block 7

In the last 12 months, have you taken HIV pre-exposure prophylaxis (PrEP) to protect yourself from HIV?

- ◯ No
- ◯ Yes
- ◯ I do not know

End of Block: Block 7

Start of Block: Block 8

Where did you hear about this survey?

- ○ Adelaide Sexual Health Centre (ASHC)
- ○ ASHM Health
- ○ a[test] (Sydney)
- ○ Canberra Sexual Health Centre (CSHC)
- ○ Centre Clinic
- ○ Clinic 16 – St Leonards
- ○ East Canberra General Practice
- ○ Gold Coast Sexual Health Service
- ○ Jack'd
- ○ M Clinic WAAC (Perth)
- ○ Melbourne Sexual Health Centre (MSHC)
- ○ Metro North Health
- ○ North Coast NSW Clinics
- ○ Ochre Medical Centre Bruce

○ Prahran Market Clinic

○ RAPID Clinic (Queensland)

○ Sexual Health Service Alice Springs – Clinic 34

○ Sydney Sexual Health Centre (SSHC)

○ HIV/LGBTIQA+ community-controlled organisations (e.g. ACON, Thorne Harbour Health)

○ Friends

○ Scruff

○ Social media (e.g. Facebook, Twitter/X)

○ South Melbourne Doctors

○ South Terrace Clinic

○ View Street Medical

○ Other (please specify) ____

End of Block: Block 8

Start of Block: Block 9

Section B. Doxycycline post-exposure prophylaxis or Doxy-PEP. ***In this section, we will ask you about your experience and/or views about doxycycline post-exposure prophylaxis. Note: Please refer to 'Doxy-PEP' as doxycycline post-exposure prophylaxis for STI prevention in this section.***

Have you heard about taking an antibiotic (doxycycline) after sex to prevent getting an STI? e.g. Doxycycline post-exposure prophylaxis (or Doxy-PEP)

○ No

○ Yes

○ Unsure

JS

What do you think about DoxyPEP?

____

End of Block: Block 9

Start of Block: Section C

Section C. Sexual Health. *In this section, we will ask you about your sexual practices and sexual health.*

What is your current relationship status?

◯ Single and sexually active

◯ Not sexually active

◯ In a sexual relationship with one or more regular partner(s), including partners, boyfriend, husband (Please ignore if your relationship is non-sexual)

◯ Prefer not to answer

◯ Other (Please specify) ____

In the last 12 months, how many sexual partners have you had sex with? *(Please enter a number)*

____

JS

Do you like Doxy-PEP?

____

End of Block: Section C

Start of Block: Block 26

In the last 12 months, which sexually transmitted infection(s) have you been diagnosed with? *(Please select all that apply)*

- ☐ Chlamydia
- ☐ Genital warts
- ☐ Gonorrhoea
- ☐ Herpes
- ☐ HIV
- ☐ Mpox (monkeypox)
- ☐ Mycoplasma genitalium (MGen)
- ☐ Syphilis
- ☐ Other (please specify) ____________________
- ☐ ⊗ I don't know
- ☐ ⊗ Prefer not to answer
- ☐ ⊗ Have not been diagnosed with an STI in the last 12 months

End of Block: Block 26

Start of Block: Block 27

In the last 12 months, have you attended a sex party or orgy?

○ No

○ Yes

○ Prefer not to answer

End of Block: Block 27

Start of Block: Block 28

In the last 12 months, have you had casual sex while you were travelling on a holiday?

○ No

○ Yes

○ Prefer not to answer

End of Block: Block 28

Start of Block: Block 29

Have you had any of the following vaccines? (Please select all that apply)

- ☐ COVID-19 vaccine
- ☐ Flu vaccine
- ☐ Hepatitis A/B vaccine
- ☐ Human papillomavirus (HPV) vaccine
- ☐ Meningococcal vaccine
- ☐ Mpox (monkeypox) vaccine
- ☐ ⊗None of the above
- ☐ ⊗I do not know

End of Block: Block 29

Start of Block: Section D

Section D. Prize Draw.

Just one more question to check before the prize draw, what was the survey about that you have just finished? *(This helps us filter out automated responses and verify participation)*

○ ABCDE survey

○ Antibiotics or doxycycline post-exposure prophylaxis survey

○ This is not a survey

○ I do not know

End of Block: Section D

Start of Block: Block 31

Thank you for completing the survey. Would you like to enter the prize draw to win one (1) of five (5) $200 Visa gift cards?

○ No

○ Yes (you will be redirected to a separate survey to enter your contact details)

End of Block: Block 31

Start of Block: Block 10

Could you please tell us where you heard about Doxy-PEP from? *(Please select all that apply)*

- ☐ Healthcare professionals (e.g. doctors)
- ☐ HIV/LGBTIQA+ community-controlled organisations
- ☐ Online community group or forum (e.g. Facebook)
- ☐ Friends
- ☐ Sex partners
- ☐ Media (e.g. social media, news)
- ☐ Online providers of Doxy-PEP
- ☐ Other (please specify): ____

End of Block: Block 10

Start of Block: Block 33

Who do you trust most to provide you with information about DoxyPEP? *(Please rank your top three choices from 1-3 by entering the number, with 1 being the most trusted)*

______ Healthcare professionals (e.g. doctors)

______ Peer community organisations

______ Online community group or forum (e.g. Facebook)

______ Friends

______ Sex partners

______ Media (e.g. social media, news)

______ Online providers of Doxy-PEP

______ Other (please specify):

End of Block: Block 33

Start of Block: Block 32

Have you ever used Doxy-PEP?

○ No

○ Yes

○ Prefer not to answer

End of Block: Block 32

Start of Block: Block 11

*

In the last 12 months, how many days did you take one or more doxycycline tablets for Doxy-PEP? *(Please enter a number)*

____

How did you usually take Doxy-PEP?

○ 100mg as a single dose after sex

○ 200mg as a single dose after sex

○ 100mg every day

○ Other method (please specify): ____

How soon after sex did you usually take Doxy-PEP?

○ Within 24 hours after sex

○ Within 48 hours after sex

○ Within 72 hours after sex

○ Within a week after sex

○ I take it every day

○ Prefer not to answer

End of Block: Block 11

Start of Block: Block 12

What was the reason(s) for you deciding to use Doxy-PEP? *(Please select all that apply)*

- ☐ Reduce my risk of getting an STI from my partner(s)
- ☐ Reduce the risk of passing an STI to my regular sex partner(s)
- ☐ Reduce the risk of passing an STI to my casual sex partner(s)
- ☐ Reduce the need for STI treatment, such as an injection to treat gonorrhoea or syphilis
- ☐ Reduce how often I need to get tested for STIs
- ☐ I think my risk of getting an STI is high
- ☐ Recommended by a doctor or health professional
- ☐ My partner did not want to use a condom
- ☐ Other (please specify): ____

Which infections were you hoping to prevent by taking Doxy-PEP? *(Please select all that apply)*

- ☐ Chlamydia
- ☐ Gonorrhoea
- ☐ Genital warts
- ☐ Herpes
- ☐ HIV
- ☐ Mpox (monkeypox)
- ☐ Mycoplasma genitalium (MGen)
- ☐ Syphilis
- ☐ Other (please specify) ____

End of Block: Block 12

Start of Block: Block 13

Do you have any concerns about taking Doxy-PEP? (Please select all that apply)

- ☐ I do not have any concerns about taking Doxy-PEP.
- ☐ I cannot afford the cost including the medication and doctor's consultations.
- ☐ I am worried about me getting antibiotic resistance while taking it (i.e. the antibiotics I take no longer cure infections I get).
- ☐ I am worried about increasing the risk of antibiotic resistance in my community (i.e. antibiotics used to treat infections in the community are no longer effective).
- ☐ I am worried about the side effects of doxycycline.
- ☐ I did not remember to take Doxy-PEP around the time of sex.
- ☐ Other (Please specify) ____
- ☐ ⊗ I do not have any concerns.

End of Block: Block 13

Start of Block: Block 14

Since you started using Doxy-PEP, have you changed how often you test for sexually transmitted infections?

- ◯ I test less frequently than before.
- ◯ I test more frequently than before.
- ◯ About the same frequency.

End of Block: Block 14

Start of Block: Block 15

Have you ever given or received Doxy-PEP with anyone else? i.e. sharing Doxy-PEP with anyone?

- ◯ No
- ◯ Yes
- ◯ Prefer not to answer

End of Block: Block 15

Start of Block: Block 16

Where did you get your Doxy-PEP? *(Please select all that apply)*

- ☐ Directly from a GP/doctor in Australia (in-person or telehealth consult)
- ☐ From a local pharmacy with a prescription
- ☐ Online with prescription
- ☐ Online without prescription
- ☐ Overseas (in person) with a prescription. Please specify the overseas country:
____
- ☐ Overseas (in person) without a prescription. Please specify the overseas country:
____
- ☐ From a friend or sex partner
- ☐ From a trial or study
- ☐ Left over from another treatment
- ☐ Other (please specify): ____

End of Block: Block 16

Start of Block: Block 17

You have indicated that you obtained Doxy-PEP online, where did you purchase it online?

- ○ Please list the website here: ____

- ○ I cannot remember which website

End of Block: Block 17

Start of Block: Block 18

What was the reason(s) for you **NOT** using Doxy-PEP? *(Please select all that apply)*

- ☐ I think my risk of getting an STI is low.
- ☐ I cannot afford the costs including the antibiotic and doctor’s consultations.
- ☐ I have difficulties in getting the antibiotic.
- ☐ I have difficulties in getting a prescription.
- ☐ I am worried that I will develop antibiotic resistance (i.e. the antibiotics I take no longer cure infections I get).
- ☐ I am worried about increasing the risk of antibiotic resistance in my community (i.e. antibiotics used to treat infections in the community are no longer effective).
- ☐ I am worried about any side effects from doxycycline.
- ☐ I am allergic to doxycycline or have previously had a bad reaction to doxycycline.
- ☐ I do not want people to know I am taking Doxy-PEP.
- ☐ Not enough information for me to decide if I should take Doxy-PEP.
- ☐ Other (please specify) ____

End of Block: Block 18

Start of Block: Block 19

Have you used any antibiotics other than doxycycline to prevent getting an STI? If you only take antibiotics as treatment, please select ‘No’. *(Please select all that apply)*

- ◯ No
- ◯ Yes
- ◯ Unsure

End of Block: Block 19

Start of Block: Block 20

You indicated that you have used other antibiotics to prevent STIs other than doxycycline, which antibiotics have you used? *(Please select all that apply)*

- ☐ Amoxicillin
- ☐ Azithromycin
- ☐ Ceftriaxone
- ☐ Ciprofloxacin
- ☐ Erythromycin
- ☐ Penicillin
- ☐ Other (please specify): ____
- ☐ ⊗I do not know

End of Block: Block 20

Start of Block: Block 21

In the next 12 months, do you plan to use Doxy-PEP?

◯ No

◯ Yes

◯ Unsure

End of Block: Block 21

Start of Block: Block 22

Which infections are you hoping to prevent by taking Doxy-PEP? *(Please select all that apply)*

- ☐ Chlamydia
- ☐ Gonorrhoea
- ☐ Genital warts
- ☐ Herpes
- ☐ HIV
- ☐ Mpox (monkeypox)
- ☐ Mycoplasma genitalium (MGen)
- ☐ Syphilis
- ☐ Other (please specify) ____

End of Block: Block 22

Start of Block: Block 23

On a scale of 0 to 10, where 0 is no benefit and 10 is the maximum benefit, how much do you think Doxy-PEP would benefit your community? Benefits may include improving sexual health and well-being within your community.

- ○ 0
- ○ 1
- ○ 2
- ○ 3
- ○ 4
- ○ 5
- ○ 6
- ○ 7
- ○ 8
- ○ 9
- ○ 10

Could you please tell us what was the factor(s) or reason(s) behind this rating? (Optional)

____

End of Block: Block 23
